# The Brain NGF Metabolic Pathway in Health and in Alzheimer’s Pathology

**DOI:** 10.3389/fnins.2019.00062

**Published:** 2019-02-12

**Authors:** A. Claudio Cuello, Rowan Pentz, Hélène Hall

**Affiliations:** ^1^Department of Pharmacology and Therapeutics, McGill University, Montreal, QC, Canada; ^2^Department of Neurology and Neurosurgery, McGill University, Montreal, QC, Canada; ^3^Department of Anatomy and Cell Biology, McGill University, Montreal, QC, Canada

**Keywords:** nerve growth factor, cholinergic system, Alzheimer’s disease, basal forebrain cholinergic nuclei, trophic support

## Abstract

Emerging research has re-emphasized the role of the cortical cholinergic system in the symptomology and progression of Alzheimer’s disease (AD). Basal forebrain (BF) cholinergic nuclei depend on target-derived NGF for survival during development and for the maintenance of a classical cholinergic phenotype during adulthood. In AD, BF cholinergic neurons lose their cholinergic phenotype and function, suggesting an impairment in NGF-mediated trophic support. We propose that alterations to the enzymatic pathway that controls the maturation of proNGF to mature NGF and the latter’s ulterior degradation underlie this pathological process. Indeed, the NGF metabolic pathway has been demonstrated to be impaired in AD and other amyloid pathologies, and pharmacological manipulation of NGF metabolism has consequences *in vivo* for both levels of proNGF/NGF and the phenotype of BF cholinergic neurons. The NGF pathway may also have potential as a biomarker of cognitive decline in AD, as its changes can predict future cognitive decline in patients with Down syndrome as they develop preclinical Alzheimer’s pathology. New evidence suggests that the cholinergic system, and by extension NGF, may have a greater role in the progression of AD than previously realized, as changes to the BF precede and predict changes to the entorhinal cortex, as anticholinergic drugs increase odds of developing AD, and as the use of donepezil can reduce rates of hippocampal and cortical thinning. These findings suggest that new, more sophisticated cholinergic therapies should be capable of preserving the basal forebrain thus having profound positive effects as treatments for AD.

## Introduction

The extraordinary discovery of nerve growth factor (NGF) by Rita Levi Montalcini and Stan Cohen, under the umbrella of Victor Hamburger’s laboratory (Cohen et al., 1954; Newmark, 1986; Cowan, 2001), not only brought a Nobel Prize to Rita Levi-Montalcini and Stan Cohen but also opened the way to the discovery of many other trophic factors and their receptors. This family plays a very significant role in the development and function of peripheral and central nervous system (CNS) neurons, and critically, some trophic mechanisms appear altered in neurodegenerative conditions. This brief review focuses on the significance of NGF for the maintenance of the cholinergic phenotype in the mature and fully differentiated CNS. It discusses the existence of a metabolic pathway explaining the release of the NGF precursor molecule and its maturation and degradation in the extracellular space. Furthermore, we discuss the compromise of this pathway in Alzheimer’s disease (AD), explaining the well-established atrophy of NGF-dependent cholinergic neurons of the basal forebrain (BF), and the possibility of deriving accessible biomarkers of AD in preclinical stages based on the NGF metabolic deregulation. Lastly, we discuss possible therapeutic interventions to normalize the NGF metabolism in AD, thus protecting the viability of the cortical and hippocampal “cholinergic tone.”

## NGF is Responsible for the Maintenance of the Cholinergic Phenotype

Nerve growth factor has been clearly implicated in the survival of basal forebrain cholinergic neurons (BFCN) in development (Gnahn et al., 1983; Large et al., 1986; Mobley et al., 1986; Auburger et al., 1987; Crowley et al., 1994; Li et al., 1995; Fagan et al., 1997; Avignone et al., 1998; Tomioka et al., 2014) but not in the fully differentiated and mature CNS. In adulthood the presence of NGF is essential for the maintenance of the BFCN phenotype (Cuello, 1993) but not for their survival, even after extreme lesions of NGF-releasing nerve terminal sites (Sofroniew et al., 1990). The first evidence for NGF-reparative effects in the adult CNS came from studies applying axotomy of the fimbria fornix projection to deprive BFCN of target-derived NGF, which led to an apparent neuronal loss of cholinergic neurons of the medial septum (Hefti, 1986; Williams et al., 1986; Kromer, 1987).

In 1982, Whitehouse and collaborators proposed that the cortical cholinergic biochemical depletion in AD was due to the loss of magnocellular (presumed cholinergic) neurons of the nucleus basalis, based on Nissl studies in which only the large (magnocellular) neurons were counted (Whitehouse et al., 1982). Later assessments of choline acetyltransferase (ChAT) immunoreactivity in rodents demonstrated that large cortical stroke lesions, which eliminated cholinergic nerve terminals, resulted not in cell loss but rather in a loss of size and cholinergic character of the cholinergic neurons of the nucleus basalis (Sofroniew et al., 1983; Cuello et al., 1986). Furthermore, it was demonstrated that a similar atrophy of ChAT-immunoreactive neurons, rather than cell loss, was observed in the human nucleus basalis of Meynert in AD (Pearson et al., 1983). These observations led us to propose that in AD, the primary pathology results in a secondary retrograde atrophy of cholinergic neurons of the nucleus basalis (Cuello and Sofroniew, 1984) which could be rescued by the application of exogenous mature NGF (mNGF), including the compensatory *de novo* cholinergic synaptogenesis of the remaining, non-lesioned, cortical tissue.

The most definitive experimental evidence for neuronal atrophy following NGF deprivation came from the excitotoxic elimination of NGF-producing neurons in the hippocampus, sparing cholinergic nerve terminals but nonetheless inducing a similar atrophy of NGF-dependent neurons of the BF (Sofroniew et al., 1990).

A number of thorough experimental studies confirmed the ability of exogenous NGF (both recombinant and isolated from the maxillary gland) to support NGF–dependent cholinergic nuclei of the BF (nucleus basalis and medial septum) following their disconnection from the sites of NGF production (Sofroniew et al., 1983; Sofroniew and Pearson, 1985; Stephens et al., 1985; Kromer, 1987; Hagg et al., 1988; Cuello et al., 1989, 1992; Koliatsos et al., 1990, 1991; Tuszynski et al., 1990; Fischer and Björklund, 1991; Maysinger et al., 1993; Garofalo and Cuello, 1994, 1995; Burgos et al., 1995; Hu et al., 1997) and also in models of aging (Fischer et al., 1987). Importantly, it was demonstrated that exogenous NGF could elicit a compensatory *de novo* cholinergic synaptogenesis in the remaining non-lesioned cortical tissue in the mature and fully differentiated CNS (Garofalo et al., 1992).

Our lab introduced the concept that the day-to-day expression of endogenous mNGF regulates the steady-state number of cortical cholinergic synapses (Debeir et al., 1999) and, in consequence, the maintenance of the “cholinergic tone.” This is in line with the classical Hebbian notion that synaptic growth is a brain activity-dependent phenomenon (Hebb, 1949). Indeed, altering the availability of endogenous NGF by pharmacologically blocking its conversion from proNGF to mNGF or by preventing its degradation does lead to notable changes in the density of cortical cholinergic terminals (Allard et al., 2012) as well as in the size and phenotype of BF cholinergic cell bodies (Allard et al., 2018).

The transcription of major cholinergic markers has been shown to be dependent on the signaling of the NGF ligand through the NGF receptor TrkA; this includes the expression of TrkA itself (Venero et al., 1994; Figueiredo et al., 1995) as well as the acetylcholine synthesis enzyme ChAT and the vesicular acetylcholine transporter VAChT (Gnahn et al., 1983; Stephens et al., 1985; Hartikka and Hefti, 1988; Pongrac and Rylett, 1998; Berse et al., 1999; Madziar et al., 2005), which share a common locus and transcriptional regulation and are often considered together as the “cholinergic gene locus.” *In vitro* experiments have shown that the ability of NGF to upregulate ChAT expression in BFCNs is enhanced by administration of gangliosides (Cuello et al., 1989) or with the co-culture of glial cells (Takei et al., 1988). Lastly, the extent of dendritic arbors, axon length, and the characteristic multipolarity of BF cholinergic cells have all been shown to be dependent on NGF (Hartikka and Hefti, 1988; Markova and Isaev, 1992).

Central to this process seems to be the homeobox transcription factor LIM homeobox 8, or Lhx8, the expression of which is essential for the development of BFCN (Mori et al., 2004). Lhx8 directly controls the expression of TrkA, is essential for normal release of acetylcholine, and is induced by NGF signaling through the ERK pathway (Tomioka et al., 2014). As ChAT and VAChT are downstream of TrkA, Lhx8 may function as a NGF-responsive master regulator of cholinergic character, both in development and in the adult organism.

## Possible Clinical Application of Exogenous NGF

As discussed above, there is abundant experimental literature supporting the ability of “exogenous” mNGF to recover atrophic BFCN in rodent lesion models as well as in non-human primates. Such strong experimental evidence provoked a number of clinical attempts to apply exogenous mNGF in the cerebroventricular space of AD patients (Jönhagen et al., 1998), attempts which were discontinued due to adverse off-target effects, specifically the induction of back pain by the sprouting of pain-conducting fibers in the spinal cord dorsal root ganglia.

Most recently, attempts to apply NGF therapeutically in the context of AD have employed gene therapy to overexpress the NGF gene (Tuszynski et al., 2005, 2015; Rafii et al., 2014) or the implantation of microencapsulated patient-derived fibroblasts, genetically engineered to overproduce NGF (Eriksdotter-Jönhagen et al., 2012; Eyjolfsdottir et al., 2016). These have achieved some success, demonstrating, variously, increases in BFCN arborization, cortical glucose metabolism, and nicotinic binding as well as normalization of cholinergic markers in the CSF, and inconsistent reports of reductions in cognitive decline (Tuszynski et al., 2005, 2015; Eriksdotter-Jönhagen et al., 2012; Ferreira et al., 2015; Karami et al., 2015; Eyjolfsdottir et al., 2016). Ultimately, the gene therapy approach was discontinued after a Phase 2 study which, though well tolerated, demonstrated no effect on cognitive outcomes (Rafii et al., 2014), while the use of microencapsulated engineered fibroblasts awaits definitive assessments of clinical efficacy (see also “Innovative Therapy for AD” by Maria Eriksdotter in this issue).

While these approaches show great promise, the application of exogenous NGF will inevitably entail the risk of off-target effects. Trophic NGF signaling is tightly constrained both in space (synaptic compartmentalization) and in time (activity dependence). NGF applied outside these parameters will not necessarily have the desired effect. Furthermore, any interruptions in NGF trafficking or excessive degradation of NGF in AD would not necessarily be overcome by exogenous application. An ideal approach would be to rather enhance the availability of endogenous NGF specifically at its physiological sites of synthesis, release, and signaling.

## Atrophy of BFCNs in AD and its Causes and the NGF Paradox

It is well established that BFCN are highly vulnerable to AD pathology, as they are observed in greatly reduced numbers at clinical presentation (Whitehouse et al., 1981). As discussed above, these neurons were initially presumed to have died (Whitehouse et al., 1981, 1982) but were later shown to persist in an atrophic state. The nucleus basalis of Meynert may be preferentially vulnerable compared to the medial septum and diagonal band (Mufson et al., 1989). The atrophy of the BFCN in AD correlates to disease duration (Mufson et al., 1989), amyloid load (Kerbler et al., 2015), and to cognitive status (Grothe et al., 2010), demonstrating that BFCN degeneration is tightly linked to amyloid accumulation and cognitive decline in early clinical AD. These findings, in combination with the downregulation of cholinergic markers in AD and the amnesic effects of anticholinergic compounds in aged individuals (Drachman and Leavitt, 1974), led Bartus to formulate the much-quoted Cholinergic Hypothesis of Geriatric Memory Dysfunction (Bartus et al., 1982).

The cholinergic hypothesis has been mischaracterized in the intervening years, as researchers sought a contrast to the well-supported and etiological amyloid hypothesis despite the fact that Coyle et al. (1983) made it clear in a Science review that the “Cholinergic Hypothesis of AD” meant a significant involvement of the cholinergic system in the pathology, and not causality. At about the same time, in a TINS review, we raised the possibility that the cholinergic atrophy could be secondary to a cortical lesion in AD (Cuello and Sofroniew, 1984) and Bartus et al. (1985) revised the status of “The Cholinergic Hypothesis,” clarifying further that “it states nothing about etiological factors” but rather describes the role of cholinergic dysfunction in memory mechanisms. Nonetheless, the cholinergic hypothesis continues to be maligned as having “failed to cure AD.” The cholinergic hypothesis led to the development of treatments, which have shown temporary cognitive benefits in patients (Adlimoghaddam et al., 2018; Birks and Harvey, 2018; Hampel et al., 2018). It is remarkable, given the widespread and irreversible brain damage already present at AD clinical presentation, that cholinergic therapy can still exert these beneficial cognitive effects.

After the publication of Bartus’s paper, it was reasonably assumed that there should be a trophic NGF failure (Appel, 1981; Hefti, 1983) to explain the cholinergic deficit in AD, an issue provoking much attention at the time. However, paradoxically, it was shown that NGF mRNA is unchanged in AD (Goedert et al., 1986), and that NGF immunoreactivity is elevated (Crutcher et al., 1993; Scott et al., 1995; Fahnestock et al., 1996). When it was later demonstrated that the signal previously measured as NGF was in fact proNGF, i.e., the NGF precursor molecule (Fahnestock et al., 2001; Peng et al., 2004), which massively predominates in the brain, it remained an open question why the BF should deteriorate when the precursor to its trophic ligand is elevated.

## A Novel NGF Metabolic Pathway and its Pharmacological Validation

**FIGURE 1:**
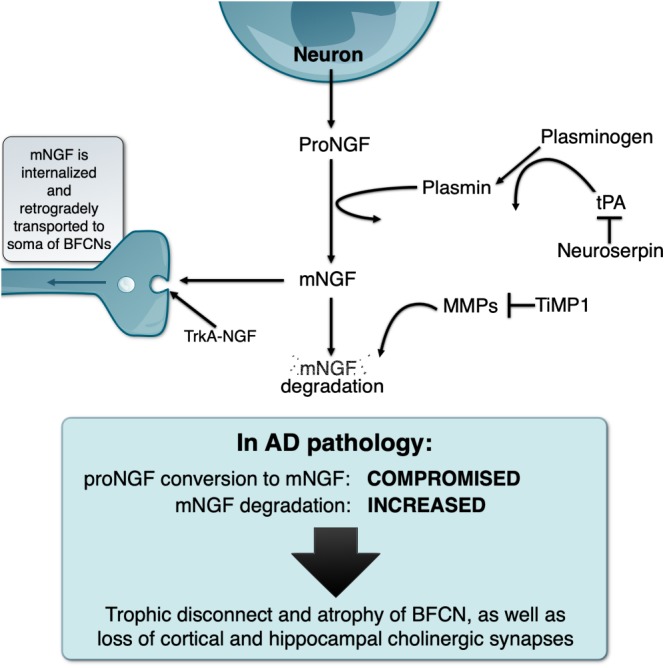
Schematic representation of the NGF metabolic pathway under physiological conditions and its deregulation in the Alzheimer’s pathology, explaining the “trophic disconnect” of NGF-dependent cholinergic neurons of the basal forebrain.

In light of the above, our laboratory investigated how mNGF was generated and released in the extracellular space by performing classical *ex vivo* superfusion of young rat cerebral cortex tissue (Bruno and Cuello, 2006). To our surprise, we found that, contrary to the prevalent dogma, proNGF (and not mNGF) was the molecular form released in an activity-dependent manner by cortical neurons *ex vivo.* We found that released proNGF is converted to mNGF and ultimately degraded by metalloproteases in the extracellular space. The conversion of proNGF into mNGF was found to be performed by plasmin, a serine-protease derived from plasminogen when cleaved by tissue plasminogen activator (tPA). This process is regulated by neuroserpin, the main inhibitor of tPA in the target regions of the BF cholinergic system (Krueger et al., 1997). Newly formed mNGF exists only transiently in the extracellular space as it binds to its cognate receptors (tropomysin receptor kinase A [TrkA] and p75NTR receptors) and is retrogradely transported to the cholinergic neurons cell somata where it exerts its trophic actions (Seiler and Schwab, 1984; Grimes et al., 1996, 1997; Figure 1).

We demonstrated that the non-internalized, extracellular remnants are likely to be degraded by activated matrix-metalloprotease 9 (MMP-9), a process controlled by tissue inhibitor of metalloproteinases 1 (TIMP-1). We also showed that the enzymes, zymogens, regulators, and inhibitors necessary for proNGF maturation and subsequent mNGF degradation are released simultaneously alongside proNGF in the extracellular space upon neuronal stimulation. The term NGF metabolic pathway was therefore coined to refer to this coordinated array of enzymatic conversions (Bruno and Cuello, 2006; Figure 1).

We further validated this pathway by demonstrating that pharmacological inhibition of the maturation or degradation enzymatic pathway can trigger proNGF/mNGF imbalances (Allard et al., 2012, 2018). In particular, continuous infusion of α2-antiplasmin, the endogenous inhibitor of plasmin, in the cerebral cortex of rats led to reduced levels of mNGF, which further led to cortical cholinergic synaptic atrophy. Conversely, infusion of MMP2-9 inhibitor induced an increase in mNGF levels by preventing its degradation, which was associated with cholinergic synaptic sprouting (Allard et al., 2012), thereby confirming the important role of the endogenous mNGF in the day-to-day maintenance of cholinergic synapses. In a further study, impaired proNGF cortical maturation following infusion of α2-antiplasmin was sufficient to alter BFCN phenotype. In particular, there was a significant atrophy of cell bodies as well as a downregulation of p75NTR and TrkA, and a reduction in the expression of ChAT protein, further validating the importance of the extracellular NGF metabolic pathway for the phenotypic maintenance of BFCN (Allard et al., 2018).

The experimental studies highlighted above reinforce the concept that imbalances in the NGF metabolic pathway can contribute to impairment of the BF cholinergic system, as seen in AD and down syndrome (DS), thereby providing a platform to investigate AD pathogenesis through the NGF pathway perspective.

## The NGF Metabolic Pathway in AD and in DS With AD Pathology

Following the discovery and validation of the NGF metabolic pathway, we applied this paradigm to the study of human brain material in AD and DS, both of which exhibit a marked atrophy of the NGF-dependent BF cholinergic system. In AD brains, proNGF levels are increased (Peng et al., 2004) while NGF biosynthesis is normal (Goedert et al., 1986). However, we demonstrated that there is in fact a compromise in the conversion of proNGF to biologically active mNGF as well as a likely exacerbated degradation of mNGF (Bruno et al., 2009a), therefore explaining that a trophic failure is responsible for the cholinergic atrophy in AD. In addition, at the mild cognitive impairment (MCI) stage (a prodromal stage of AD), the pathway is already compromised and shows increased MMP9 activity, which would facilitate the degradation of mNGF (Bruno et al., 2009b). This would suggest that the NGF dysfunction appears early in the course of the disease, before or concurrent with the earliest cognitive impairment and may constitute a relevant therapeutic target.

As individuals with DS inexorably develop AD neuropathology (Head and Lott, 2004), we investigated in DS genetic models, fetal cortical cells and post-mortem brain samples, whether the AD pathology in DS impaired the NGF metabolic pathway. These studies eloquently illustrated a compromise of the NGF metabolic pathway responsible for the conversion of proNGF to mNGF in DS brains with AD pathology. Levels of plasmin and tPA mRNA were reduced, preventing proNGF maturation and resulting in the pathological brain build-up of proNGF. DS brains also exhibited elevated zymogenic activity of MMP9, thereby exacerbating the degradation of the limited amount of available biologically active mNGF (Iulita et al., 2014). These results have been further reviewed in (Iulita and Cuello, 2016; Iulita et al., 2016a).

In a further extension, levels of key members of the NGF metabolic cascade, inflammatory mediators and Aβ peptides were assessed in plasma samples from a clinically characterized cohort of DS individuals, longitudinally followed for 2 years. Levels of proNGF, MMP-1 and MMP-3, and MMP-9 activity were even elevated at AD asymptomatic stages. In addition, this study provided compelling evidence that DS/AD asymptomatic individuals showing elevation of plasma proNGF levels at the 1-year follow-up experienced a greater cognitive deterioration the subsequent year. Furthermore, in DS individuals of advanced age there was a strong correlation between brain amyloid-beta (Aβ) load and the elevation of proNGF, strengthening the association between Aβ and NGF metabolic pathway dysfunction (Iulita et al., 2016b). A similar progressive deregulation of the NGF metabolic pathway has been found in transgenic rats modeling the AD-like amyloid pathology (Iulita et al., 2017; Figure 1).

## Novel Developments Regarding the Implication of the Cholinergic System in AD

New developments, such as the ones highlighted above, have led to a renewed interest regarding the neurobiology of the BF cholinergic system in health and in neurodegenerative conditions. Recent observations note that long term treatment with donepezil, one of the leading acetylcholine esterase inhibitors (AChEis) (elevating the “cholinergic tone”) used for its symptomatic cognitive benefits in AD, was shown to reduce the rate of change in regional cortical thickness in individuals suspected of prodromal AD (Cavedo et al., 2016) as well as diminish the rate of hippocampal (Dubois et al., 2015) and BF atrophy in prodromal AD (Cavedo et al., 2017).

Conversely, recent studies provide evidence of brain atrophy and increased risk of dementia and AD in non-demented individuals receiving long term (over 3 years) medications with drugs possessing primary or secondary anti-cholinergic properties (Jessen et al., 2010; Gray and Hanlon, 2016; Risacher et al., 2016; Chuang et al., 2017). In particular, there is consistent evidence for increased risk of dementia in the elderly receiving defined anticholinergic compounds (Gray and Hanlon, 2018). In addition, low doses of the muscarinic antagonist scopolamine leads to poor cognitive performance in individuals revealing *a posteriori* AD-like brain amyloid burden (Snyder et al., 2014, 2015).

It is also interesting to note that a study from the Alzheimer’s disease neuroimaging initiative (ADNI) revealed that in humans the atrophy of the BF nucleus basalis of Meynert precedes and predicts memory impairment and the extent of cortical spread of the Alzheimer’s pathology (Schmitz et al., 2016). In further extensions, these studies indicate that atrophy of the BF covaries with the cortical thinning and loss of PET-identified cholinergic projections to cortical regions (Schmitz et al., 2018) and that BF volume predicts rate of global cognitive deterioration better than hippocampal volume (Teipel et al., 2018). These studies therefore reaffirm that the BFCN are central to the AD pathology and its cognitive outcomes. This concept is frequently questioned by the fact that cholinergic therapy only provides modest and transient cognitive relief in AD. As such, it should be noted that novel drugs targeting the cholinergic system, beyond the cholinesterase inhibitors approved for the symptomatic treatment of AD, are emerging as therapeutic tools, in particular muscarinic receptor agonists (Fisher, 2012; Verma et al., 2018). In particular, a combined M1 muscarinic-sigma 1 receptor was shown to have a remarkable effect in diminishing amyloid pathology and the associated cognitive impairment in a transgenic rat model of AD-like amyloid pathology, even after a treatment “wash-out” of a month (equivalent to 3 years of human life) (Hall et al., 2018).

The above strongly suggest that trophic preservation of BF cholinergic neurons (i.e., normalization of the NGF metabolic pathway) could constitute an effective avenue to achieve disease-modifying effects. An appropriate therapeutic window for such an approach should lie at pre-symptomatic stages of AD, before the cholinergic system is compromised beyond repair. Such goals await the development of biomarkers defining unequivocally a progressing AD pathology before its clinical presentation.

## Conclusion and Future Challenges

This discussion paper highlights the significance of the NGF metabolic pathway for the maintenance of a healthy phenotype of BFCN and synapses, a system key for higher CNS functions. It illustrates how the compromise of this pathway likely underlies the atrophy of BFCN in AD (see Figure 1).

Recent clinical data would indicate that these trophic mechanisms might not necessarily be unidirectional, as the current NGF dogma indicates. Future studies may reveal novel insights into possible reciprocal interactions between the BF cholinergic system and the NGF metabolic pathway, both in physiological and pathological conditions. Further to it, research on such trophic interactions could help identify novel biomarkers signaling a progressive preclinical AD pathology and pave the way for novel therapeutic interventions for AD and associated pathologies with NGF cholinergic involvement.

## Author Contributions

ACC, RP, and HH wrote and edited the manuscript. All authors approved it for publication.

## Conflict of Interest Statement

The authors declare that the research was conducted in the absence of any commercial or financial relationships that could be construed as a potential conflict of interest.
